# Contrast Agent Pooling in the Descending Aorta Due to Severe Heart Failure

**DOI:** 10.5811/cpcem.20340

**Published:** 2024-11-01

**Authors:** Yosuke Maezawa, Kazuya Nagasaki, Hiroyuki Kobayashi, Shunsuke Sakai, Toshiyuki Irie

**Affiliations:** * Mito Kyodo General Hospital, Department of Internal Medicine, University of Tsukuba, Mito, Japan; † Mito Kyodo General Hospital, Department of Cardiology, University of Tsukuba, Mito, Japan; ‡ Mito Kyodo General Hospital, Department of Radiology, University of Tsukuba, Mito, Japan

**Keywords:** heart failure, contrast agent pooling, aortic dissection

## Abstract

**Case presentation:**

An 86-year-old female presented to our emergency department with chest pain and orthopnea and was diagnosed with heart failure and ST-elevation myocardial infarction, prompting hospitalization. During hospitalization, she developed a fever. A chest and abdominal contrast-enhanced computed tomography (CT), conducted to investigate the cause of the fever, coincidentally revealed sedimentation of contrast agent in the descending aorta. To differentiate from aortic dissection, we conducted dynamic CT, and it was confirmed that the contrast agent within the aorta decreased over time. On the same day, an echocardiogram revealed a left ventricular ejection fraction of 36% with reduced contractile function, and a stagnant, hazy echo within the descending aorta.

**Discussion:**

In aortic dissection, the retention of contrast agent in the false lumen of the aorta is a crucial finding for diagnosis. However, we experienced a case where contrast agent accumulated in the descending aorta, caused by low ejection fraction of the left ventricle. Differential diagnosis from aortic dissection may be possible due to the gradual decrease in contrast agent over time. This case is valuable to report given the limited number of previous reports on this phenomenon.

## CASE PRESENTATION

An 86-year-old female with no remarkable medical history except for untreated and uncontrolled hypertension presented with bilateral lower leg edema for two weeks. She had palpitations starting a week prior. She complained of chest pain and orthopnea in the early morning, and she was subsequently transported to our hospital. She was diagnosed with heart failure and ST-elevation myocardial infarction involving the lateral wall, prompting hospitalization. Percutaneous coronary intervention (PCI) was considered but was initially declined by the patient. The decision was made to focus on heart failure management first and reconsider PCI after heart failure improvement. Non-invasive positive pressure ventilation was initiated, along with nitroglycerin, hydralazine, and furosemide, improving her respiratory distress. On the fourth day of hospitalization, oxygen therapy was discontinued, and on the fifth day all treatments were transitioned from intravenous to oral administration.

On the eighth day of hospitalization, she developed a fever. A chest and abdominal contrast-enhanced computed tomography (CT), conducted to investigate the source of the fever, did not reveal any apparent focus of infection. During the portal venous phase of the contrast-enhanced CT, sedimentation of contrast agent (CA) in the descending aorta was observed, suggesting a decrease in aortic flow velocity. To differentiate from aortic dissection, dynamic CT was performed. As the arterial, portal venous, and equilibrium phases were sequentially acquired, it was confirmed that the CA within the aorta decreased over time (Image).

On the same day as the CT, an echocardiogram was performed, revealing a left ventricular ejection fraction of 36% with reduced contractile function. Diffuse hypokinesis was noted, particularly with decreased wall motion in the inferolateral-inferior wall. There was dilation of the ascending aorta, and we observed moderate aortic and mitral valve regurgitation. No enlargement of the right heart chambers, thrombi, or vegetations were observed. The blood flow velocity in the descending aorta varied due to atrial fibrillation but was generally around 50–80 centimeters per second. Echography revealed a stagnant, hazy echo within the descending aorta.

## DISCUSSION

Here we report CT images showing CA retention due to decreased aortic flow velocity in severe heart failure. In cases of cardiac arrest or cardiogenic shock, CA pooling in the venous system in CT images has been reported as “contrast agent pooling sign.”^1^,^2^ To our knowledge, there have been no reports of such findings of aortic involvement, particularly in those not requiring inotropic support or oxygen supplementation.

**CPC-EM Capsule**

What do we already know about this clinical entity?

*Contrast agent pooling is linked to cardiogenic shock or cardiopulmonary arrest, but its association with low-output heart failure has not been reported.*

What is the major impact of the image(s)?

*Our case shows dynamic CT can detect changes in contrast agent pooling, ruling out aortic dissection.*

How might this improve emergency medicine practice?

*Dynamic CT is essential for diagnosing and differentiating between aortic dissection and contrast agent pooling conditions such as low-output heart failure.*

We concluded that the pooling of CA was due to low cardiac output syndrome, resulting from the reduced ejection fraction and regurgitation. The retention of CA in the aorta on CT, particularly in patients with heart failure, strongly suggests reduced cardiac output. Although aortic dissection is a critical differential diagnosis that could cause such findings on CT, the gradual change in CA pooling observed in dynamic CT can help differentiate between these two conditions.

## Figures and Tables

**Image:**
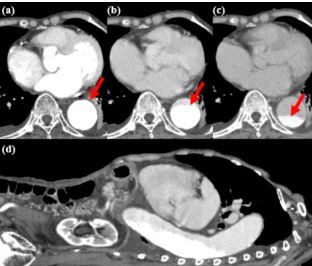
Dynamic, contrast-enhanced computed tomography of the chest and abdomen,demonstrating lessening contrast in aorta over time (arrows): (a) axial image 30 seconds after contrast agent injection; (b) 90 seconds after injection; and (c) 240 seconds after injection; (d) sagittal image at 90 seconds.
